# Effects of Bitter Melon and a Chromium Propionate Complex on Symptoms of Insulin Resistance and Type 2 Diabetes in Rat Models

**DOI:** 10.1007/s12011-020-02202-y

**Published:** 2020-06-02

**Authors:** Pandora E. White, Ewelina Król, Artur Szwengiel, Małgorzata Tubacka, Dawid Szczepankiewicz, Halina Staniek, John B. Vincent, Zbigniew Krejpcio

**Affiliations:** 1 Department of Chemistry and Biochemistry, The University of Alabama, Tuscaloosa, AL 35487-0336 USA; 2 Institute of Human Nutrition and Dietetics, The Poznań University of Life Sciences, Wojska Polskiego 31, 60-624 Poznań, Poland; 3 Institute of Food Technology of Plant Origin, The Poznań University of Life Sciences, Wojska Polskiego 31, 60-624 Poznań, Poland; 4 Department of Animal Physiology and Biochemistry, The Poznań University of Life Sciences, Wolynska 35, 60-637 Poznań, Poland

**Keywords:** Chromium, Bitter melon, Rat, Diabetes, Insulin resistance

## Abstract

Trivalent chromium (Cr) and bitter melon (*Momordica charantia* L., BM) have been shown to independently interact with the insulin signaling pathway leading to improvements in the symptoms of insulin resistance and diabetes in some animal models and human subjects. The aim of this study was to examine whether the combination of the two nutritional supplements could potentially have additive effects on treating these conditions in high-fat-fed streptozotocin (STZ)-induced diabetic rats. The experiment was conducted with 110 male Wistar rats divided into eleven groups and fed either a control or high-fat diet for 7 weeks. Half of the rats on the high-fat diet were injected with STZ (30 mg/kg body mass) to induce diabetes. The high-fat (HF) diets were then supplemented with a combination of Cr (as chromium(III) propionate complex, Cr3: either 10 or 50 mg Cr/kg diet) and bitter melon (lyophilized whole fruit: either 10 or 50 g/kg diet) for 6 weeks. After termination of the experiment, blood and internal organs were harvested for blood biochemical, hematological, and mineral (Cr) analyses using appropriate analytical methods. It was found that neither Cr(III) nor BM was able to significantly affect blood indices in HF and diabetic rats, but BM tended to improve body mass gain, blood glucose, and LDL cholesterol values, but decreased Cr content in the liver and kidneys of the Cr-co-supplemented type 2 diabetic model of rats. Supplementary Cr(III) had no appreciable effect on glucose and lipid metabolism in high-fat-fed STZ-induced diabetic rats. Supplementary BM fruit powder had some observable effects on body mass of high-fat-fed rats; these effects seem to be dampened when BM was co-administered with Cr. Cr(III) and BM appear to act as nutritional antagonists when both administered in food, probably due to binding of Cr by the polyphenol-type compounds present in the plant material.

**Figure:**
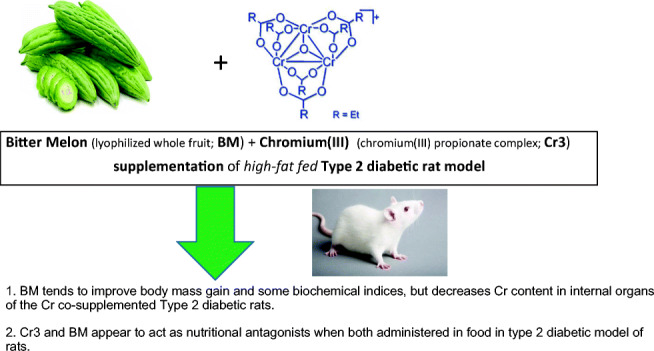
Graphical Abstract

Graphical Abstract

## Introduction

Type 2 diabetes is characterized by a decline in β cell function and worsening insulin resistance. Several decades ago, chromium (Cr), as its trivalent ion, was shown to enhance glucose uptake in rat fat cells. Since then, Cr has been used as a “micronutrient” in multivitamin formulations, parenteral nutrition, food, and energy drinks. However, the mechanism by which Cr works remains unclear, although the mechanism is pharmacologically rather than nutritionally relevant, requiring supra-nutrition doses for beneficial effects to be observed [1]. One of the most studied Cr(III) supplements is the cation [Cr_3_O(propionate)_6_(H_2_O)_3_]^+^ or Cr3 (also commonly called chromium propionate) [2–7]. The effects of Cr3 on healthy rats and rat models of insulin resistance and type 1 and type 2 diabetes have been examined where it was found to have beneficial effects on insulin sensitivity and, in most cases, lipid parameters [8–15]. Alternatively, *Momordica charantia*, commonly known as bitter melon (BM), has been used in Asia and some parts of Africa as a prophylactic against diabetes [16–21]. The beneficial effects of BM may be attributed to phytonutrients having antidiabetic properties such as charantin, vicine, and polypeptide-p as well as other unspecific phytochemicals [16, 20, 21]. The potential use of bitter melon–derived compounds as antidiabetics has been recently discussed in the article by Elekofehint et al. [21], thus will not be repeated in this work.

The world market of dietary supplements is a lucrative multi-billion dollar business. Some complex supplement formulas contain various phytochemicals or selected fractions derived from fruits, vegetables, or medicinal plants, being often combined with vitamins and minerals, including trace elements. The purpose of the present study was to examine whether the combined Cr(III) and bitter melon have an effect on insulin resistance and type 2 diabetes in high-fat-fed streptozotocin (STZ)-induced diabetic rats.

## Experimental Methods

### Animals

A total of 110 Wistar rats approximately 8 weeks old were obtained from the Licensed Laboratory Animal Breeding Center (Poznań, Poland). During both the adaption and the experimental periods, the animals were housed under controlled temperature (21 ± 2 °C), humidity (55–60%), and with a 12-h/12-h day/night cycle. After a 1-week acclimation period, the animals were assigned to one of 11 experiment groups of 10 rats each. The rats were pair-housed in metal cages (covered with ceramic metal–free enamel) without bedding throughout the study. The animals had ad libitum access to drinking water and prepared feed. Animals were weighed weekly. Feed was weighed daily. The experiment was carried out with approval of the Institutional Animal Care and Use Committee of The University of Alabama (approval no. 16-05-011) and the Animal Bioethics Committee of Poznań, Poland (approval #no. 62/2016 9.09.2016).

### Materials

The source of supplemental Cr(III) was [Cr_3_O(propionate)_6_(H_2_O)_3_]^+^ nitrate, also called Cr3. Cr3 was synthesized following the method described previously by Earnshaw et al. [22]. Bitter melon (*Momordica charantia* L.) plant was grown in the experimental field at Poznań University of Life Science, Poland, in the summer season (2015). The mature whole fruit of bitter melon, after collection, was rinsed in tap water, air-dried, cut up into slices, lyophilized, ground into powder, and kept in plastic bags in a freezer. The chemical composition of the materials is presented in Table 2. Streptozotocin was obtained from Sigma-Aldrich (St. Louis, MO, USA).

### Insulin Resistance and Diabetic Model

All rats received either a standard or high-fat diet for 48 days depending on group assignment. The high-fat diet was utilized to induce insulin resistance (IR). To induce type 2 diabetes, rats were fed a high-fat diet for 48 days, followed by a single intraperitoneal injection with 30 mg streptozotocin (STZ, dissolved in citric buffer pH 4.5)/kg body mass. Three days post injection, blood was collected from tail slits, and blood glucose was measured using a glucometer (Genexo iXell). The rats were considered diabetic if glucose levels were ≥ 180 mg/dL.

### Rat Diets

The eleven experimental groups and corresponding diets were designated as control (C), high-fat diet control (HF), high fat supplemented with low dose of Cr(III) and low dose of BM (HF+Cr1BM1), high fat supplemented with low dose of Cr(III) and high dose of BM (HF+Cr1BM2), high fat supplemented with high dose of Cr(III) and low dose of BM (HF+Cr2BM1), high fat supplemented with high dose of Cr(III) and high dose of BM (HF+Cr2BM2), diabetic control (Db), diabetic supplemented with low dose of Cr(III) and low dose of BM (Db+Cr1BM1), diabetic supplemented with low dose of Cr(III) and high dose of BM (Db+Cr1BM2), diabetic supplemented with high dose of Cr and low dose of BM (Db+Cr2BM1), and diabetic supplemented with high dose of Cr(III) and high dose of BM (Db+Cr2BM2). The high-fat diet was based on AIN-93 diet (Table 1(A)) but with partial replacement of wheat starch (Table 1(B)). Cr(III) and bitter melon (BM) powder were administered to appropriate groups daily via feed for the remainder of the study, after the determination of diabetes status for the diabetic rats. Cr(III) and BM were used as partial replacements of wheat starch in the high-fat diet. The diets with a low dose of Cr(III) contained 10 mg Cr/kg diet, while the high-dose diets contained 50 mg Cr/kg diet.

**Table 1:** Diet compositions

Ingredient	%	g/kg
(A) Composition of control diet		
Casein	14	140
Sunflower oil	4.0	40
Wheat starch	62.2	622
Sucrose	10.0	100
Potato starch	5.0	50
Mineral mix*	3.5	35
Vitamin mix	1.0	10
l-Cysteine	0.3	3
Sum	100	1000

(B) Composition of high-fat diet		
Casein	14	140
Sunflower oil	4.0	40
Lard	16.0	160
Wheat starch	46.2	462
Sucrose	10.0	100
Potato starch	5.0	50
Mineral mix	3.5	35
Vitamin mix	1.0	10
l-Cysteine	0.3	3
Sum	100	1000

*Mineral mix composed according to AIN-93G recommendations

The diets with a low dose of BM contained 10 g BM/kg diet, while the high-dose diets contained 50 g BM/kg diet.

The doses of supplemental Cr(III) (given as Cr3) were determined to mimic supra-nutritional (or pharmacologic) doses, based on previous experiments performed in our lab [23–26], at 10 and 50 mg of Cr/kg diet, equal to 1 and 5 mg Cr/kg body mass (low and high levels). Similarly, the doses of supplemental bitter melon (BM) were determined to mimic supra-nutritional (or pharmacologic) doses, based on previous studies [27–29], at 10 and 50 g BM/kg diet (1% and 5% diet), equal to 1 and 5 g BM/kg body mass (low and high levels).

The composition of the bitter melon dry powder is given in Table 2.

**Table 2:** Chemical composition of bitter melon

Component	Mean	SD
Ash (%)	10.19	0.41
Protein	11.90	1.77
Fat (%)	2.15	0.20
Carbohydrates (%)	66.19	1.92
Dry matter (%)	90.11	0.49
Ca (mg/100 g)	333.09	77.14
Mg (mg/100 g)	199.90	17.99
Fe (mg/100 g)	6.23	0.92
Zn (mg/100 g)	2.970	0.063
Cu (mg/100 g)	0.598	0.097
Quercetin (mg/100 g)	2.067	0.44
Quercetin glucoside (mg/100 g)	3.083	1.82
Peltatoside (mg/100 g)	5.675	1.54
Charantoside (mg/100 g)	1.535	1.39

### Blood and Tissue Sampling

After 90 days, all animals were sacrificed, and blood samples were taken from the heart. Blood was allowed to clot for 2 h before centrifuging for 10 min. Serum was stored at − 70 °C. The liver, kidney, spleen, heart, testes, and pancreas were harvested. All were washed with 0.9% NaCl solution. Portions of the pancreas, liver, and kidney were placed in 4% formalin for histopathy assays. The rest of the samples were snap-frozen in liquid nitrogen and stored at − 70 °C.

These were centrifuged at 100,000×*g* for 10 min. Final body mass and body length were also measured. Epididymal fat and skeletal muscle from the femur were also harvested, flash-frozen, and stored at − 70 °C until being shipped to the USA to be used for biochemical assays.

### Enzyme-Linked Immunosorbent Assays

Enzyme-linked immunosorbent assays (ELISA) were performed to quantify the extent of phosphorylation of protein kinase B (Akt) or insulin receptor substrate 1 (IRS-1). Portions of the skeletal muscle were homogenized in extraction buffer prepared according to Cefalu and co-workers [30]. The extracts were centrifuged at 100,000×*g* at 25 °C for 10 min to remove insoluble components. The supernatant was used for assays. In order to standardize the amount of protein used in the ELISA, protein concentration was determined using a commercial bicinchoninic acid (BCA) protein assay (Thermo Scientific, Rockford, IL, USA).

The amount of total Akt and phosphorylated Akt was determined using a commercially available ELISA kit for detection and quantification of rat Akt and phosphorylated Akt (Sigma-Aldrich, St. Louis, MO, USA). The amount of phosphorylated IRS-1 was determined using a commercially available ELISA kit for phosphorylated IRS-1 (Cell Signaling, Danvers, MA, USA). Total IRS-1 was determined using a commercially available ELISA kit for pan-IRS-1 (Cell Signaling, Danvers, MA, USA).

### Blood Biochemistry

Red blood cells (RBC), hemocrit, mean corpuscular volume (MCV), mean corpuscular hemoglobin (MCH), mean cell hemoglobin concentration (MCHC), and white blood cells (WBC) were analyzed using the CELLDYN-1700 analytical hematology system. Hemoglobin (HGB) concentration was determined by the Drabkin cyanmethemoglobin method. Serum glucose concentration was determined by the hexokinase method, while insulin concentration was measured by the radioimmunoassay (RIA) method (Linco Research, St. Charles, MO, USA). Total cholesterol, HDL, LDL, and triglycerides levels were determined using the Olympus AU 560 equipment by colorimetric methods [31, 32]. Alanine aminotransferase (ALT), aspartate aminotransferase (AST), and alkaline phosphatase (ALP) levels were measured by kinetic methods [33]. Total protein concentration was measured by the biuret method [34], while creatinine and urea concentrations were measured by the kinetic method using urease and glutamine dehydrogenase and the Jaffe kinetic method with picric acid [35], respectively.

The efficacy of glucose utilization and insulin resistance was characterized by the homeostasis model assessment (HOMA) indices as calculated using Eq. 1 proposed by Antunes et al. [36].

1 $$ \mathrm{HOMA}-\mathrm{IR}=\frac{\left(\mathrm{fasting}\ \mathrm{glucose}\ \left[\mathrm{mmol}/{\mathrm{dm}}^3\right]\times \mathrm{fasting}\ \mathrm{insulin}\ \left[\mathrm{mIU}/{\mathrm{dm}}^3\right]\right)}{22.5} $$

### Phenolic Compounds and Charantoside Determination

In brief, 500 mg of lyophilized sample was extracted using 16 mL of 80:20 (v/v) methanol/H_2_O. Then, suspensions were sonicated at room temperature for 30 min, and thereafter the mixture was centrifuged at 4000 rpm for 15 min and the supernatant was collected. After centrifugation, the solvent was evaporated to dryness. The residue was dissolved in 3.5 mL (3 mL MeOH + 0.5 mL H_2_O) and finally, the supernatant was filtered with a PTFE syringe filter (0.45-μm pore size). Phenolic compounds were determined by reversed-phase (RP) ultrahigh-performance liquid chromatography (Dionex UltiMate 3000 UHPLC, Thermo Fisher Scientific, Sunnyvale, CA, USA) coupled with electrospray ionization mass spectrometry (ESI-MS) with an ultrahigh-resolution orthogonal quadrupole time-of-flight (qTOF) accelerator (maXis impact, Bruker Daltonik, Bremen, Germany). The RP-UHPLC separation was performed using C18 column in positive and negative MS mode.

### Atomic Absorption Spectrometry

Samples of the testes, pancreas, kidney, liver, heart, and spleen were used for atomic absorption spectrometry. The organs were digested in 65% (w/w) spectra pure HNO_3_. Cr concentrations were determined by graphite furnace analysis using a Hitachi ZA 3000 atomic absorption spectrophotometer (Hitachi High-Technologies Corporation, Tokyo, Japan). The accuracy of quantitative determinations of Cr was confirmed by a simultaneous analysis of the certified reference material (Pig Kidney BCR® No. 186, Brussels, fortified with the Cr standard).

### Statistics

Data were analyzed by the Kruskal-Wallis one-way analysis of variance (ANOVA) followed by a Dunn’s method pairwise multiple comparisons procedure to determine specific significant differences (*p* ≤ 0.05) using SigmaPlot 11 (SPSS, Inc., Chicago, IL). When appropriate, data are presented in figures and tables as means ± standard deviation (SD). Some important parameters (e.g., HOMA-IR, serum insulin levels) with non-linear distribution were log-transformed to assure a normal distribution pattern. Interactions between Cr(III) and BM were analyzed by the two-way ANOVA followed by Bonferroni’s test using SigmaPlot 11 (SPSS, Inc., Chicago, IL) to determine if there were significant interactions (*p* ≤ 0.05).

## Results and Discussion

### General Growth Indices

**Fig. 1:**
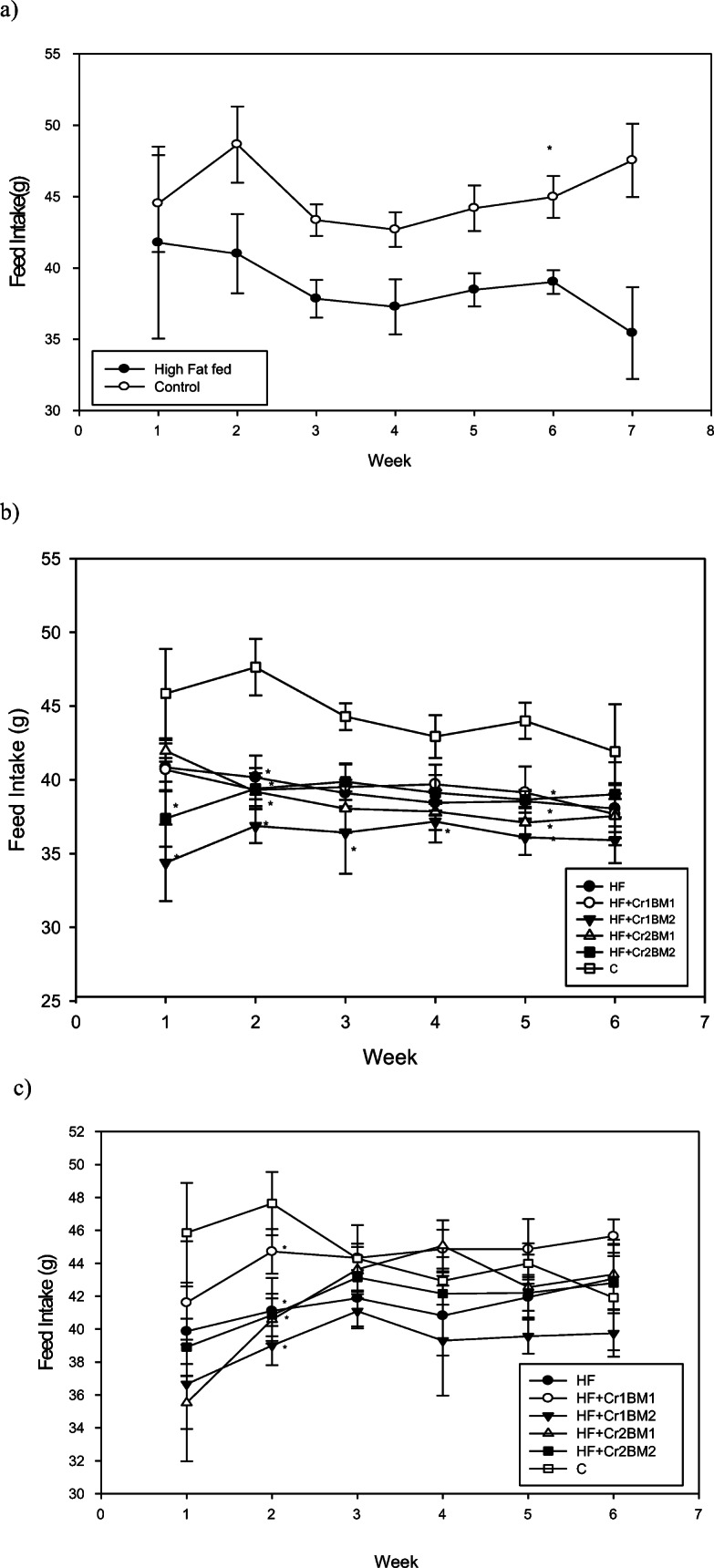
Feed intake (g/day/rat) for rats. **a** Control rats and rats on HF diet for the first 7 weeks. **b** Control and insulin-resistant rats for 6 weeks of treatment after initial 7 weeks on control or HF diet. **c** Control rats and diabetic rats for 6 weeks of treatment after initial 7 weeks on control or HF diet

**Fig. 2:**
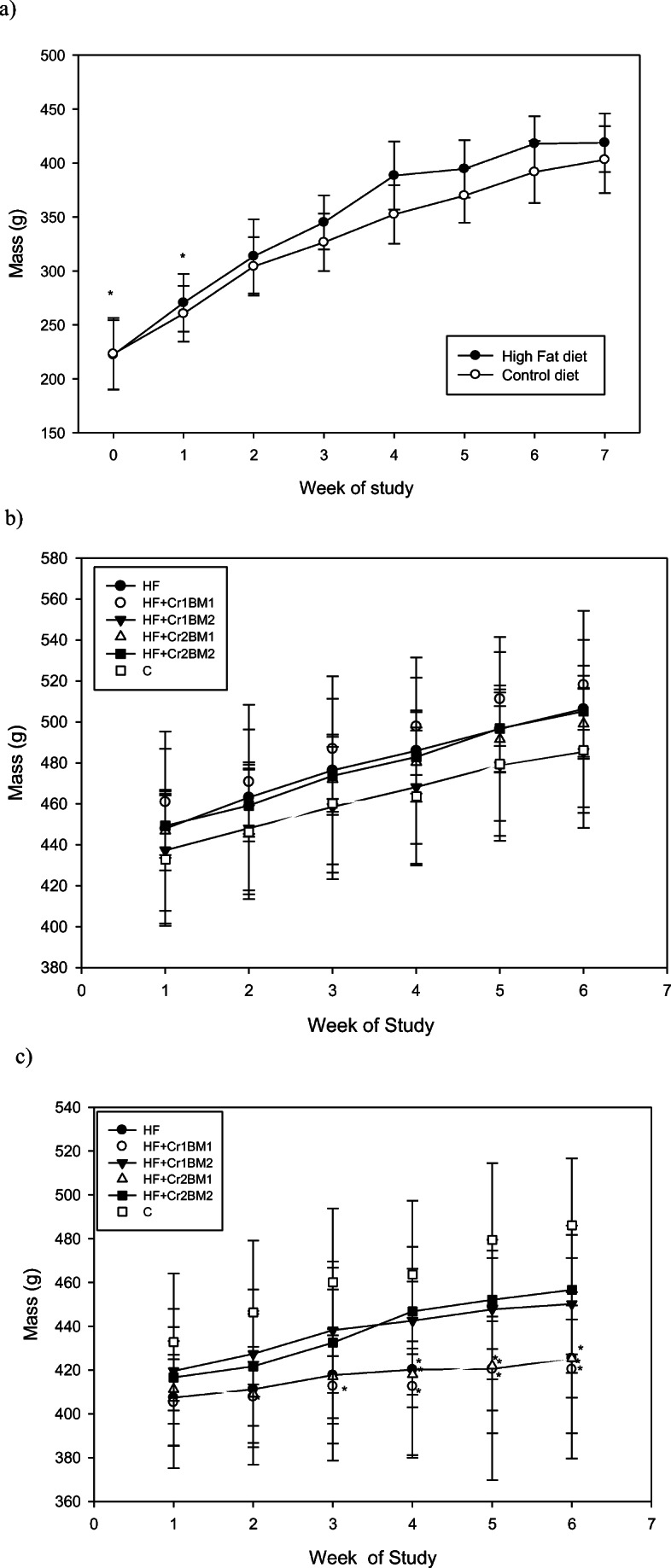
Body mass (g/rat) of rats. **a** Control rats and rats on high-fat diet for the first 7 weeks. **b** Control and insulin-resistant rats for 6 weeks of treatment after initial 7 weeks on control or high-fat diet. **c** Control rats and diabetic rats for 6 weeks of treatment after initial 7 weeks on control or high-fat diet. Asterisk symbol indicates statistically significantly different than control on a given week

Feed intake of rats fed HF diet from weeks 2 to 7 tended to be lower than that of control rats while body mass gain was statistically significantly higher than that of the control group (Figs. 1a and 2a). Past studies have also shown that rats fed a HF diet possessed increased body mass as compared with control groups [23]. After introduction of Cr(III) and BM to the appropriate groups of rats, the insulin-resistant rats fed the HF diet still had lower food intakes and tended to have less body mass, although the latter were not statistically significant. No effects were observed on these variables from the introduction of Cr(III) or BM (Figs. 1b and 2b). Several studies have shown that Cr(III) does not affect body mass [8, 9, 14, 15, 23–26, 37–40]. A reduction of body mass of HF-fed rodents due to treatment of BM has been reported previously [41, 42]. For the diabetic rats, no effects on food intake were generally observed (Fig. 1c); however, these rats had a significant reduction in body mass compared with the control rats (Fig. 2c). Cr(III) had no effect and the low dose of BM had no effect on body mass, while a high dose of BM, regardless of the associated dose of Cr(III), significantly restored body mass, so body mass of these rats was statistically equivalent to that of the control (Fig. 2c). Thus, BM appears to have a dramatic effect in preventing body mass loss associated with HF- and STZ-induced diabetes.

Table 3 shows growth indices, including body length and internal organ masses. The spleen, heart, testes, and pancreas masses were not significantly different between the groups. The diabetic rats, however, tended to have more massive kidneys than the insulin-resistant or control rats, while the higher dose of BM tended to partially restore the increased mass. A similar but weaker trend can be observed for the livers of the diabetic rats, but none of the differences is statistically significant for the liver masses. Thus, BM at the higher dose may potentially have some protective effects on the liver and kidneys, although studies with larger populations of rats may be required to resolve this issue. Yoon et al. also found a significant reduction of liver mass for HF mice treated with bitter melon extract [42]; however, not all studies have observed an effect of organ masses, in particular liver mass, including one that used freeze-dried BM [43]. Chromium(III) has not been found to have reproducible effects on body composition or organ mass previously [1]. In particular, Cr3 did not affect body composition or tissue masses of male Wistar rats on a high-fat diet or fat diet in combination with STZ treatment [23, 25].

**Table 3:** Effect of diet and supplemental Cr and BM on overall growth indices in rats (mean ± SD)

Parameter	Experimental group										
	C	Db	Db+Cr1BM1	Db+Cr1BM2	Db+Cr2BM1	Db+Cr2BM2	HF	HF+Cr1BM1	HF+Cr1BM2	HF+Cr2BM1	HF+Cr2BM2
Body length ratio (cm)	26.55 ± 4.46	25.70 ± 7.13	25.55 ± 6.81	26.00 ± 6.65	25.56 ± 6.48	26.00 ± 6.09	26.40 ± 5.93	26.70 ± 5.70	25.70 ± 7.13	25.55 ± 6.81	26.00 ± 6.65
Liver (g)	13.70 **±** 1.32	16.00 **±** 1.87	15.33 **±** 2.06	14.82 **±** 1.94	16.10 **±** 2.60	14.70 **±** 1.41	14.04 **±** 1.63	14.03 **± ±**1.85	13.02 **±** 1.48^a,c^	13.53 **±** 0.86	14.18 **±** 1.90
Kidneys (g)	2.864 **±** 0.21^c^	3.311 **±** 0.36	3.301 **±** 0.44	3.010 **±** 0.38	3.517 **±** 0.35	3.027 **±** 0.39	2.698 **±** 0.38^a,b,c^	2.948 **±** 0.41^c^	2.727 **±** 0.23^a,b,c^	2.808 **±** 0.13^c^	2.828 **±** 0.24^c^
Spleen (g)	0.635 **±** 0.07	0.587 **±** 0.07	0.579 **±** 0.06	0.599 **±** 0.05	0.558 **±** 0.08	0.617 **±** 0.07	0.597 **±** 0.08	0.609 **±** 0.09	0.547 **±** 0.04	0.601 **±** 0.05	0.590 **±** 0.03
Heart (g)	1.287 **±** 0.16	1.180 **±** 0.09	1.189 **±** 0.14	1.164 **±** 0.07	1.193 **±** 0.09	1.195 **±** 0.10	1.239 **±** 0.15	1.298 **±** 0.12	1.239 **±** 0.12	1.283 **±** 0.10	1.243 **±** 0.13
Testes (g)	3.888 **±** 0.29	3.716 **±** 0.24	3.509 **±** 0.40	3.737 **±** 0.29	3.652 **±** 0.18	3.726 **±** 0.19	3.829 **±** 0.29	3.890 **±** 0.22	3.788 **±** 0.17	3.827 **±** 0.23	3.704 **±** 0.14
Pancreas (g)	2.11 **±** 0.30	2.139 **±** 0.14	2.084 **±** 0.12	2.187 **±** 0.22	2.027 **±** 0.17	2.135 **±** 0.19	2.230 **±** 0.16	2.276 **±** 0.11	2.227 **±** 0.19	2.299 **±** 0.17	2.273 **±** 0.11

^a^Significantly different from Db-HF

^b^Significantly different from Db-Cr1BM1

^c^Significantly different from Db-Cr2BM1

### Blood Biochemistry Indices Related to Glucose and Lipid Metabolism

Cr(III) (as Cr3) has previously been shown to improve insulin sensitivity and blood cholesterol levels in healthy rats and several rat models of insulin resistance and type 2 diabetes, including male Wistar rats on a high-fat diet and treated with STZ which had improved HOMA-IR and reduced levels of triglycerides and total and LDL cholesterol from Cr3 supplementation [23]. However, not all studies have found beneficial effects as effects seem to be less significant in female rats [37, 39] and male Wistar rats on a high-fat diet had no effects from Cr3 on serum cholesterol, glucose, or insulin concentrations [23]. Thus, the diabetic rats in this study should serve as a positive control for effects from Cr3, while the insulin-resistant rats should serve as a negative control in terms of effects on insulin resistance and serum cholesterol.

Bitter melon has been shown to improve glucose levels and lipid profile in insulin-resistant rodents [41–44]. For example, Yoon et al. demonstrated bitter melon extract reduced LDL and total cholesterol, glucose, and insulin in HF mice treated with BM for 12 weeks as compared with HF controls [42]. Wang et al. found plasma glucose, insulin, and HOMA-IR were significantly lowered in HF C57Bl/J mice treated with BM extract for 12 weeks as compared with HF controls [41].

**Fig. 3:**
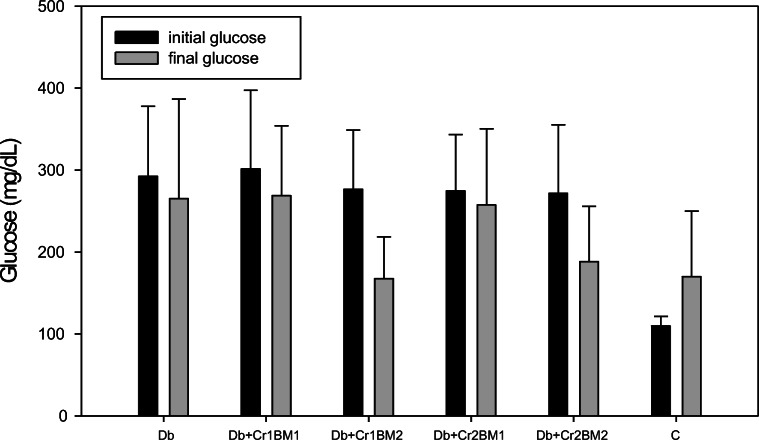
Serum glucose levels (mg/dL) of control and diabetic rats. Initial glucose represents blood glucose at the start of the Cr and BM treatment. Final serum glucose is the glucose levels after 6 weeks of treatment with Cr and BM

In the current study, serum glucose, insulin, total cholesterol, LDL cholesterol, HDL cholesterol, and triglyceride concentrations and HOMA-IR for rats treated with HF, Cr(III), or BM were not significantly different from the values of these variables in the control group. However, a few trends were observed for these parameters among the diabetic rats (Table 4). Specifically, diabetic rats supplemented with the higher dose of BM (50 g/kg diet) tended to have lower serum glucose and LDL cholesterol levels as compared with all other treatment groups (Table 4). Similarly, the serum glucose concentrations of the diabetic rats supplemented with the higher dose of BM tended to drop compared with the serum glucose concentrations before the start of the BM administration (Fig. 3). Interestingly, the two-way ANOVA of the log-transformed HOMA-IR values (conformed normal distribution pattern, data not shown) revealed that there is a significant interaction effect between Cr(III) and BM on this parameter. The HOMA-IR values of the high-fat-fed rats supplemented with high Cr(III) dose but low BM level (HF+Cr2BM1) were significantly lower compared with the low Cr(III) dose with the same amount of BM (HF+Cr1BM1), while at high BM dose, Cr dose did not affect this parameter. Thus, the high BM dose appears to ameliorate the beneficial effect of supplementary Cr(III) on insulin resistance in high-fat-fed rats.

**Table 4:** Effect of diet and supplemental Cr and BM on blood serum variables in rats (mean ± SD)

Parameter	Experimental group										
	C	Db	Db+Cr1BM1	Db+Cr1BM2	Db+Cr2BM1	Db+Cr2BM2	HF	HF+Cr1BM1	HF+Cr1BM2	HF+Cr2BM1	HF+Cr2BM2
Final glucose concentration (mmol dm^−3^)	134.50 ± 13.4	211.41 ± 61.9	252.36 ± 73.2	167.54 ± 50.8	257.36 ± 93.1	188.26 ± 70.2	170.84 ± 50.8	140.86 ± 42.81	132.72 ± 16.4	163.35 ± 53.0	124.68 ± 15.18
Insulin concentration (mIU dm^−3^)	34.45 ± 21.0	30.87 ± 17.6	25.82 ± 10.0	17.01 ± 7.15	29.92 ± 12.25	48.14 ± 27.7	48.14 ± 27.7	43.24 ± 16.6	45.17 ± 17.5	63.32 ± 47.55	52.33 ± 21.03
HOMA-IR index	10.11 ± 6.84	16.35 ± 9.55	15.10 ± 6.64	8.89 ± 4.11	11.30 ± 5.83	12.73 ± 5.83	18.01 ± 10.9	16.22 ± 5.40	13.54 ± 4.32	15.04 ± 7.46	14.71 ± 6.84
Total cholesterol concentration (mg dm^−3^)	104.8 ± 17.5	115.2 ± 36.5	92.80 ± 24.8	98.39 ± 22.8	95.34 ± 24.3	106.2 ± 22.9	90.86 ± 22.5	86.60 ± 20.21	91.59 ± 19.1	81.35 ± 17.8	85.75 ± 15.2
HDL cholesterol concentration (mg dm^−3^)	96.44 ± 13.4	104.3 ± 38.6	87.07 ± 22.5	88.61 ± 18.4	88.09 ± 23.3	98.50 ± 26.3	83.40 ± 18.9	81.41 ± 11.22	86.90 ± 14.7	76.34 ± 16.9	77.35 ± 9.20
LDL cholesterol concentration (mg dm^−3^)	15.00 ± 5.25	14.91 ± 6.80	10.33 ± 6.20	13.14 ± 4.79	10.46 ± 4.05	13.92 ± 5.3	11.26 ± 6.40	10.35 ± 3.66	13.53 ± 4.96	9.74 ± 3.12	9.555 ± 2.86
Triglycerides (mg/dm^−3^)	151.1 ± 56.5	158.9 ± 55.6	126.1 ± 55.6	121.6 ± 79.0	121.8 ± 54.1	111.4 ± 33.7	124.5 ± 51.7	121.3 ± 67.66	107.6 ± 29.62	117.9 ± 44.5	147.97 ± 102.3

The lack of effects from Cr(III) on the diabetic rats and the limited effects from BM on the insulin-resistant rats and diabetic rats may result from an antagonistic interaction between the BM and Cr(III) (vide infra).

### Blood Biochemistry and Toxicity Indices

A blood analysis was performed to determine if any rats had any abnormalities in their blood profile (Table 5). Neither HF feeding, diabetes, nor any combination of BM and Cr(III) had an effect. Similarly, the blood was analyzed for indices of kidney and liver function (Table 6). Neither HF feeding, diabetes, nor any combination of BM and Cr(III) had an effect. For all the variables, the levels were in the reference range for healthy rats.

**Table 5:** Effect of diet and supplemental Cr and BM on blood morphology and hematology in rats (mean ± SD)

Parameter	Experimental group										
	C	Db	Db+Cr1BM1	Db+Cr1BM2	Db+Cr2BM1	Db+Cr2BM2	HF	HF+Cr1BM1	HF+Cr1BM2	HF+Cr2BM1	HF+Cr2BM2
RBC (10^12^ dm^−3^)	8.330 ± 0.43	8.554 ± 0.46	8.717 ± 0.29	8.159 ± 0.41	8.836 ± 0.26	8.562 ± 0.28	8.080 ± 0.28	8.120 ± 0.27	8.116 ± 0.28	8.192 ± 0.41	8.239 ± 0.48
HGB (mmol dm^−3^)	15.23 ± 0.55	15.44 ± 0.85	15.21 ± 0.44	15.32 ± 0.75	15.69 ± 0.72	30.37 ± 44.9	14.96 ± 0.54	15.07 ± 0.40	15.00 ± 0.48	15.14 ± 0.63	14.77 ± 0.53
Hemocrit	47.43 ± 1.82	49.67 ± 2.60	48.99 ± 1.29	47.84 ± 2.27	50.10 ± 2.46	48.37 ± 1.36	47.20 ± 1.93	47.31 ± 1.37	47.08 ± 1.47	47.63 ± 1.93	47.68 ± 3.89
MCV (10^−15^ dm^−3^)	57.00 ± 1.69	56.90 ± 1.60	56.10 ± 1.37	58.70 ± 2.83	56.90 ± 1.90	56.56 ± 1.51	58.44 ± 2.13	58.40 ± 1.65	58.00 ± 1.12	58.20 ± 2.10	56.70 ± 2.00
MCH (10^−15^ kg)	18.29 ± 0.72	18.04 ± 0.81	17.46 ± 0.45	18.78 ± 1.03b	17.73 ± 0.59	17.94 ± 0.49	18.50 ± 0.63	18.57 ± 0.42	18.48 ± 0.47	18.50 ± 0.78	17.95 ± 0.99
MCHC (10^−15^ dm^−3^)	32.09 ± 0.40	31.66 ± 0.71a	31.08 ± 0.53	31.99 ± 0.67	31.29 ± 0.40	31.80 ± 0.56	31.69 ± 0.55	31.84 ± 0.60	31.88 ± 0.61	31.78 ± 0.45	31.67 ± 0.89
WBC (10^9^ dm^−3^)	9.625 ± 5.20	5.650 ± 2.35	6.630 ± 2.03	5.580 ± 1.88	5.467 ± 0.72	7.978 ± 1.92	7.578 ± 2.22	5.730 ± 1.47	6.967 ± 2.42	6.690 ± 1.77	6.030 ± 1.09

*RBC* red blood cell count, *HGB* hemoglobin, *MCV* mean corpuscular volume, *MCH* mean corpuscular hemoglobin, *MCHC* mean corpuscular hemoglobin concentration, *WBC* white blood cell count

**Table 6:** Effect of diet and supplemental Cr and BM on blood toxicity markers in rats (mean ± SD)

Parameter	Experimental group										
	C	Db	Db+Cr1BM1	Db+Cr1BM2	Db+Cr2BM1	Db+Cr2BM2	HF	HF+Cr1BM1	HF+Cr1BM2	HF+Cr2BM1	HF+Cr2BM2
ALT (U/I)	21.18 **±** 11.9	24.33 **±** 8.24	34.39 **±** 14.4	32.62 **±** 23.8	33.69 **±** 10.3	31.62 **±** 16.0	19.67 **±** 5.40	19.29 **±** 2.28	20.05 **±** 2.89	20.51 **±** 4.68	20.95 **±** 5.06
ALP (U/I)	66.86 **±** 13.74	124.9 **±** 47.2	131.3 **±** 56.2	109.0 **±** 47.6	130.1 **±** 30.6	103.1 **±** 44.0	67.11 **±** 14.0	73.41 **±** 10.65	67.76 **±** 7.45	70.76 **±** 7.78	68.73 **±** 7.25
AST (U/I)	81.12 **±** 23.6	80.41 **±** 8.93	97.16 **±** 22.5	109.3 **±** 48.4	109.8 **±** 25.0	114.3 **±** 88.3	79.96 **±** 19.0	74.41 **±** 9.86	83.33 **±** 22.4	80.53 **±** 27.9	79.35 **±** 17.3
Total protein (10^−2^ kg dm^−3^)	66.35 **±** 4.47	62.72 6.80	59.78 **±** 6.96	62.62 **±** 5.07	59.02 **±** 6.58	63.29 **±** 6.50	63.97 **±** 2.85	63.21 **±** 2.62	64.42 **±** 3.71	64.71 **±** 4.45	63.27 **±** 3.62
Creatinine (mg dL)	0.363 **±** 0.04	0.39 **±** 0.10	0.40 **±** 0.04	0.386 **±** 0.05	0.41 **±** 0.08	0.373 **±** 0.04	0.363 **±** 0.02	0.38 **±** 0.04	0.365 **±** 0.05	0.385 **±** 0.05	0.353 **±** 0.03
Urea (mg dL)	26.34 **±** 1.30	43.73 **±** 19.4	50.77 **±** 26.0	33.22 **±** 18.5	50.77 **±** 24.3	37.65 **±** 21.1	22.52 **±** 3.47	22.88 **±** 2.71	23.97 **±** 2.88	23.23 **±** 4.14	20.54 **±** 1.56

### Tissue Cr Concentrations

Cr supplementation both in insulin-resistant and diabetic rats tended to lead to significant increases in the Cr(III) concentrations in the liver and the kidneys at both Cr(III) doses (Tables 7 and 8). For the high-fat-fed rats, the HF+Cr1BM1 rats had an appreciable increase in liver Cr concentrations compared with levels in livers of high-fat control rats; however, when the amount of dietary BM was increased fivefold for the HF+Cr1BM2 rats, the increase in liver Cr was no longer significant. For the HF rats with the higher dose of Cr(III), both the HF+Cr2BM1 and HF+Cr2BM2 rats had increased liver Cr levels compared with those of HF controls; however, the levels in HF+Cr2BM2 were significantly lower than those in HF+Cr2BM1. Hence, BM appears to lead to a reduction of Cr levels in the liver of HF rats regardless of Cr(III) dose. A similar effect is observed for kidneys of the HF+Cr2BM1 and HF+Cr2BM2 rats. For the diabetic rats, parallel effects are observed for the levels of Cr in the livers of the rats as a function of Cr(III) and BM dose. In contrast, for the kidney, the higher dose of BM resulted in a statistically significant reduction of kidney Cr at the lower dose of Cr(III), while the reduction from the higher dose of BM was not statistically significant at the higher dose of Cr(III). Thus, the higher dose of BM generally appears to prevent the accumulation of Cr in the tissues. This is probably the result of the interactions of some components of BM with Cr(III) that can bind this element in the gastrointestinal tract, lowering its absorption and deposition in the tissues. It is known that BM is rich in various phytochemicals, some of them have cation-binding properties (e.g., fractions of dietary fiber, phytates, polyphenolics, saponins, oxalates) and may have decreased the absorption of Cr(III) in the gut. BM is reported to be composed of 54.42% soluble oxalates [45]. It has been known that oxalates have been reported to interfere with the absorption of metals such as calcium, magnesium, and iron. Oxalic acid can form iron-oxalate complexes that are insoluble, making iron unavailable and inhibiting its intestinal absorption [46]. Saponins have been shown to reduce Fe uptake [18]. Polyphenols and phytates have an appreciable affinity for Fe and, thus, reduce iron uptake in the gastrointestinal tract of animals and humans [47]. In light of the results of the present study, the interaction of Cr(III) with the BM phytochemicals is hypothesized to decrease the bioavailability of Cr(III) for absorption in the gut. One possible mechanism may be first the chelation reaction of Cr(III) by polyphenols present in BM and the second one, the redox reaction between Cr(III) and BM components. The presumed decrease in Cr(III) absorption would lead to lower levels of Cr in the body, which in turn could prevent the concentrations of Cr in the tissues necessary for beneficial effects from Cr supplementation to be achieved. This would explain the lack of any beneficial effects from Cr(III) supplementation being observed in the diabetic rats receiving Cr3 and BM, while Cr3 alone had previously been found to lead to beneficial effects on insulin sensitivity and cholesterol levels in these rats [24]. In turn, if any of the chemicals in the BM that bind Cr(III) are also active in generating beneficial effects, then the presence of the Cr(III) could mitigate the effects of BM, and the beneficial effects from BM appear to be diminished against those reported in previous studies. However, most previous studies used BM extracts, which could have different bioavailability and concentrations of the bioactive species compared with the BM whole fruit crude powder used in the current study, limiting interpretation. While the relationships found in this study are statistically non-significant, they indicate tendencies and, thus, should be supported in further studies.

**Table 7:** The effects of supplementary Cr3 and BM on liver and kidney Cr levels in control and insulin-resistant rats. Values in a row with different superscript letters are statistically significantly different

Index	Experimental group					
	C	HF	HF+Cr1BM1	HF+Cr1BM2	HF+Cr2BM1	HF+Cr2BM2
Liver Cr (ng/g dry mass)	29.2 ± 12.2^a^	38.5 ± 12.2^a^	102.3 ± 12.8^b^	61.4 ± 12.2^a^	311.8 ± 12.2^d^	199.7 ± 12.8^c^
Kidney Cr (ng/g dry mass)	306.1 ± 85.8^ab^	197.8 ± 76.8^a^	534.5 ± 76.8^b,c^	555.8 ± 76.8^c^	2224.8 ± 80.9^e^	1306.8 ± 76.8^d^

**Table 8:** The effects of supplementary Cr3 and BM on liver and kidney Cr levels in control and diabetic rats. Values in a row with different superscript letters are statistically significantly different

Index	Experimental group					
	C	Db	Db+Cr1BM1	Db+Cr1BM2	Db+Cr2BM1	Db+Cr2BM2
Liver Cr (ng/g dry mass)	29.2 ± 12.2^a^	45.4 ± 26.5^a^	164.8 ± 24.8^b^	54.3 ± 24.8^a^	471.3 ± 26.3^d^	322.0 ± 23.6^c^
Kidney Cr (ng/g dry mass)	306.1 ± 85.8^a^	207.4 ± 155.4^a^	459.3 ± 164.8^a^	284.7 ± 155.4^a^	2403.2 ± 155.4^b^	2313.3 ± 147.4^b^

### Effects on Protein Phosphorylation

**Fig. 4:**
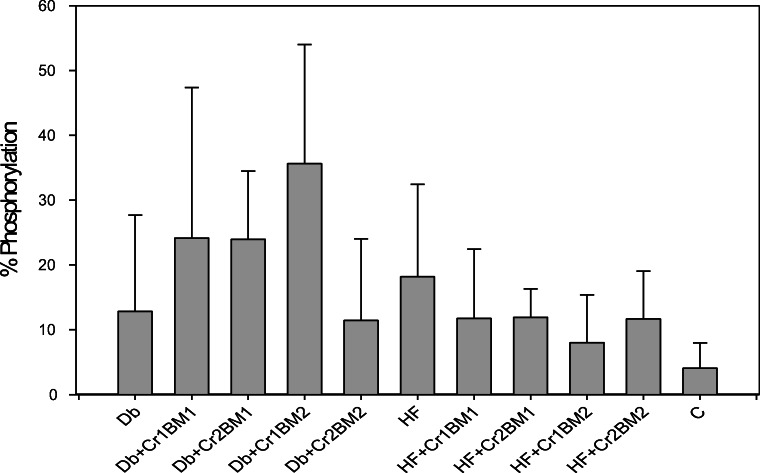
Percent IRS-1 phosphorylation after 6 weeks of treatment with Cr and BM

No significant effects from Cr(III), BM, nor HF on the phosphorylation of IRS-1 were found (Fig. 4). Cefalu et al. found insulin-stimulated IRS-1 phosphorylation increased in obese IR JCR:LA-cp rats fed Cr picolinate (80 μg Cr/kg body mass) for 3 months, although no difference was found in the absence of insulin administration [30]. In contrast, Cr3 did not increase tyrosine phosphorylation of insulin-stimulated 3T3-L1 cells [48]. In the current work, a similar lack of effects was found for the phosphorylation of Akt (data not shown). In contrast, Wang et al. found IRS-1, Akt1, and Akt 2 phosphorylation was increased in high-fat-fed C57BL/6J mice fed bitter melon extract for 12 weeks [41]. The limited bioavailability of Cr(III) and diminished activity of BM from the antagonism between the two supplements when administered together in food appear to limit the ability to probe the effects of either supplement on the insulin signaling pathway.

## Conclusions

Supplementary Cr (III) (given as Cr3), unlike in previous studies, had no appreciable effect on glucose and lipid metabolism in high-fat-fed STZ-induced diabetic rats. Supplementary BM fruit powder had some observable effects on body mass of high-fat-fed rats; these effects seem to be dampened when BM was co-administered with Cr3. Cr(III) and BM appear to act as nutritional antagonists when both administered in food, probably due to binding of Cr(III) by the polyphenol-type compounds present in the plant material.

## References

[1] Vincent JB (2013). The bioinorganic chemistry of chromium.

[2] Mulkani I, Levina A, Lay PA (2004). Biomimetic oxidation of chromium(III): does the antidiabetic activity of chromium(III) involve carcinogenic chromium(VI)?. Angew Chem Int Ed.

[3] Speetjens JK, Parand A, Crowder MW, Vincent JB (1999). Low-molecular-weight chromium-binding substance and biomimetic [Cr_3_O(O_2_CH_2_CH_3_)_6_(H_2_O)_3_]^+^ do not cleave DNA under physiologically-relevant conditions. Polyhedron.

[4] Shute AA, Vincent JB (2001). The stability of the biomimetic cation triaqua-μ-xohexapropionatotrichromium(III) in vivo in rats. Polyhedron.

[5] Davis CM, Royer AC, Vincent JB (1997). Synthetic multinuclear chromium assembly activates insulin receptor tyrosine kinase activity: functional model for low-molecular-weight chromium-binding substance. Inorg Chem.

[6] Clodfelder BJ, Chang C, Vincent JB (2004). Absorption of the biomimetic cation triqua-μ_3_-oxo-μ-hexapropionatotrichromium(III) in rats. Biol Trace Elem Res.

[7] Bailey MM, Sturdivant J, Jernigan PL, Townsend MB, Bushman J, Ankareddi I, Rasco JF, Hood RD, Vincent JB (2008). Comparison of the potential for developmental toxicity of prenatal exposure to two dietary chromium supplements, chromium picolinate and [Cr_3_O(O_2_CH_2_CH_3_)_6_(H_2_O)_3_]^+^, in mice. Birth Defects Res Develop Reprod Toxicol.

[8] Bennett R, Adams B, French A, Neggers Y, Vincent JB (2006). High-dose chromium(III) supplementation has no effects on body mass and composition while altering plasma hormone and triglycerides concentrations. Biol Trace Elem Res.

[9] Clodfelder BJ, Gullick BM, Lukaski HC, Neggers Y, Vincent JB (2005). Oral administration of [Cr_3_O(O_2_CH_2_CH_3_)_6_(H_2_O)_3_]^+^ increases insulin sensitivity and improves blood plasma variables in healthy and type 2 diabetic rats. J Biol Inorg Chem.

[10] Debski B, Krejpcio Z, Kuryl T, Wojciak R, Lipko M (2004). Biomimetic chromium(III) complex and fructan supplementation affect insulin and membrane glucose transport in rats. J Trace Elem Exp Med.

[11] Krejpcio Z, Debski B, Wojciak R, Kuryl T, Tubacka M (2004). Biomimetic chromium(III) complex and fructan supplementation improve blood variables in STZ-induced diabetic rats. J Trace Elem Exp Med.

[12] Kuryl T, Krejpcio Z, Wojciak R, Lipko M, Debski B, Staniek H (2006). Chromium(III) propionate and dietary fructans supplementation stimulate erythrocyte glucose uptake and betaoxidation in lymphocytes of rats. Biol Trace Elem Res.

[13] Pickering A, Chang C, Vincent JB (2004). Chromium-containing biomimetic cation triaqua-μ3-hexapropionatotrichromium(III) inhibits colorectal tumor formation in rats. J Inorg Biochem.

[14] Sun Y, Clodfelder BJ, Shute AA, Irvin T, Vincent JB (2002). The biomimetic [Cr_3_O(O_2_CCH_2_CH_3_)_6_(H_2_O)_3_]^+^ decreases plasma insulin, cholesterol, and triglycerides in healthy and type II diabetic rats but not type I diabetic rats. J Inorg Biochem.

[15] Sun Y, Mallya K, Ramirez J, Vincent JB (1999). The biomimetic [Cr_3_O(O_2_CCH_2_CH_3_)_6_(H_2_O)_3_]^+^ decreases plasma cholesterol and triglycerides in rats: towards chromium-containing therapeutics. J Biol Inorg Chem.

[16] Singh J, Cumming E, Manoharan G, Kalasz H, Adeghate E (2011). Medicinal chemistry of the anti-diabetic effects of Momordica charantia: active constituents and modes of actions. Open Med Chem J.

[17] Leatherdale BA, Panesar RK, Singh G, Atkins TW, Bailey CJ, Bignell AH (1981). Improvement in glucose tolerance due to Momordica charantia (karela). Brit Med J (Clinical Research Edition).

[18] Han C, Zuo J, Wang Q, Xu L, Wang Z, Dong H, Gao L (2015). Effects of 1-MCP on postharvest physiology and quality of bitter melon (Momordica charantia L.). Sci Hortic.

[19] Platel K, Shurpalekar KS, Srinivasan K (1993). Influence of bitter gourd (Momordica charantia) on growth and blood constituents in albino rats. Nahrung.

[20] Khanna P, Jain SC, Panagariya A, Dixit VP (1981). Hypoglycemic activity of polypeptide-p from a plant source. J Natural Products.

[21] Elekofehinti OO, Ariyo EO, Akinjiyan MO, Olayerijua SO, Lawal AO, Adanlawob IG, Teixeira Rocha JB (2018). Potential use of bitter melon (Momordica charantia) derived compounds as antidiabetics: in silico and in vivo studies. Pathophysiol.

[22] Earnshaw A, Figgis BN, Lewis J (1966) Chemistry of polynuclear compounds. Part VI. Magnetic properties of trimeric chromium and iron carboxylates. J Chem Society A:1656–1663

[23] Król E, Krejpcio Z, Iwanik K (2014). Supplementary chromium(III) propionate complex does not protect against insulin resistance in high-fat-fed rats. Biol Trace Elem Res.

[24] Król E, Krejpcio Z (2010). Chromium(III) propionate complex supplementation improves carbohydrate metabolism in insulin-resistance rat model. Food Chem Toxicol.

[25] Król E, Krejpcio Z (2011). Evaluation of anti-diabetic potential of chromium(III) propionate complex in high-fat diet fed and STZ injected rats. Food Chem Toxicol.

[26] Król E, Krejpcio Z, Michalak S, Wojciak RW, Bogdanski P (2012). Effects of combined dietary chromium(III) propionate complex and thiamine supplementation on insulin sensitivity, blood biochemical indices, and mineral levels in high-fructose-fed rats. Biol Trace Elem Res.

[27] Saad DY, Soliman MM, Baiomy AA, Yassin MH, El-Sawy HB (2017). Effects of karela (bitter melon; *Momordica charantia*) on genes of lipids and carbohydrates metabolism in experimental hypercholesterolemia: biochemical, molecular and histopathological study. BMC Complement Altern Med.

[28] Abas R, Othman F, Thent ZC (2015). Effect of Momordica charantia fruit extract on vascular complication in type 1 diabetic rats. EXCLI J.

[29] Yang SJ, Choi JM, Park SE, Rhee EJ, Lee WY, Oh KW, Park SW, Park CY (2015). Preventive effects of bitter melon (*Momordica charantia*) against insulin resistance and diabetes are associated with the inhibition of NF-κB and JNK pathways in high-fat-fed OLETF rats. J Nutr Biochem.

[30] Cefalu WT, Wang ZQ, Zhang XH, Baldor LC, Russell JC (2002). Oral chromium picolinate improves carbohydrate and lipid metabolism and enhances skeletal muscle Glut-4 translocation in obese, hyperinsulinemic (JCR-LA corpulent) rats. J Nutr.

[31] Yukata M (1999). A homogeneous assay for the selective measurement of LDL cholesterol in serum. Enzymatic selective protection method. Clin Lab.

[32] Shephard MD, Whiting MJ (1990). Falsely low estimation of triglycerides in lipemic plasma by the enzymatic triglyceride method with modified Trinder’s chromogen. Clin Chem.

[33] Schumann G, Klauke R (2003). New IFCC reference procedures for the determination of catalytic activity concentrations of five enzymes in serum: preliminary upper reference limits obtained in hospitalized subjects. Clin Chim Acta.

[34] Thomas L (1998) Total protein. In Thomas L (Ed.), Clinical laboratory diagnostics. Use and assessment of clinical laboratory results. TH-Books Verlagsgesellschaft, FrankfuntMain 644-647

[35] Newmann DJ, Price CP (1999) Renal function and nitrogen metabolites. In: Burtis CA, Ashwood ER (Eds.). Tietz book of clinical chemistry (3rd ed.). WB Saunders Company, Philadelphia 1239–1242

[36] Antunes LC, Elkfury JL, Jornada MN, Foletto KC, Bertoluci MC (2016). Validation of HOMA-IR in a model of insulin-resistance induced by a high-fat diet in Wistar rats. Arch Endocrin Metab.

[37] Staniek H, Krejpcio Z (2017). The effects of supplementary Cr3 (chromium(III) propionate complex) on the mineral status in healthy female rats. Biol Trace Elem Res.

[38] Staniek H, Krejpcio Z, Iwanik K (2010). Evaluation of the acute oral toxicity class of tricentric chromium(III) propionate complex in rat. Food Chem Toxicol.

[39] Staniek H, Krejpcio Z, Wieczorek D (2016). The effects of high dietary doses of chromium(III) complex with propionic acid on nutritional and selected blood indices in healthy female rats. Biol Trace Elem Res.

[40] Staniek H, Rhodes NR, Di Bona KR, Deng G, Love ST, Pledger LA, Blount J, Gomberg E, Grappe F, Cernosek C, Peoples B, Rasco JF, Krejpcio Z, Vincent JB (2013). Comparison of tissue metal concentrations in zucker lean, zucker obese, and zucker diabetic fatty rats and the effects of chromium supplementation on tissue metal concentrations. Biol Trace Elem Res.

[41] Wang ZQ, Zhang XH, Yu Y, Poulev A, Ribnicky D, Floyd ZE, Cefalu WT (2011). Bioactives from bitter melon enhance insulin signaling and modulate acyl carnitine content in skeletal muscle in high-fat diet-fed mice. J Nutr Biochem.

[42] Yoon NA, Park J, Lee J, Jeong JY, Kim H-K, Lee HS, Hwang IG, Roh GS, Kim HJ, Cho GJ, Choi WS, Lee DH, Kang SS (2017). Anti-diabetic effects of ethanol extract from bitter melon in mice fed a high-fat diet. Develop Reprod.

[43] Yang SJ, Choi JM, Park SE, Rhee EJ, Lee WJ, Oh KW, Park SW, Park CY (2015). Preventive effects of bitter melon (Momordica charantia) against insulin resistance and diabetes are associated with the inhibition of NF-κβ and JNK pathways in high-fat-fed OLETF rats. J Nutr Biochem.

[44] Sridhar MG, Vinayagamoorthi R, Arul Suyambunathan V, Bobby Z, Selvaraj N (2008). Bitter gourd (Momordica charantia) improves insulin sensitivity by increasing skeletal muscle insulin-stimulated IRS-1 tyrosine phosphorylation in high-fat-fed rats. Brit J Nutr.

[45] Bong W-C, Savage G (2018). Oxalate content of raw, wok-fried, and juice made from bitter gourd fruits. Food Sci Nutr.

[46] Kawazu Y, Okimura M, Ishii T, Yui S (2003). Varietal and seasonal differences in oxalate content of spinach. Sci Hortic.

[47] Mascitelli L, Goldstein MR (2010). Inhibition of iron absorption by polyphenols as an anti-cancer mechanism. QJM.

[48] Mackowiak P, Krejpcio Z, Sassek M, Kaczmarek P, Hertig I, Chmielewska J, Wojciechowicz T, Szczepankiewicz D, Wieczorek D, Szymusiak H, Nowak KW (2010). Evaluation of insulin binding and signaling activity of newly synthesized chromium(III) complexes in vitro. Mol Med Rep.

